# The Relationship between Secondary Forest and Environmental Factors in the Southern Taihang Mountains

**DOI:** 10.1038/s41598-017-16647-0

**Published:** 2017-11-27

**Authors:** Hui Zhao, Qi-Rui Wang, Wei Fan, Guo-Hua Song

**Affiliations:** 1 Beijing Forestry University, College of Forestry, Beijing, 100083 China; 2 Henan Academy of Forestry, Zhengzhou, 450008 China; 3 Canvard College, Beijing Technology and Business University, Beijing, 101118 China

## Abstract

It is important to understand the effects of environmental factors on secondary forest assembly for effective afforestation and vegetation restoration. We studied 24 20 m × 20 m quadrats of natural secondary forest in the southern Taihang Mountains. Canonical correspondence analysis (CCA) and two-way indicator hydrocarbon analysis were used to analyse the relationship between community vegetation and environmental factors. The CCA showed that 13 terrain and soil variables shared 68.17% of the total variance. The principal environmental variables, based on the most parsimonious CCA model, were (in order) elevation, soil total N, soil gravel content, slope, soil electrical conductivity, and pH. Samples were clustered into four forest types, with forest diversity affected by elevation, nutrients, and water gradients. Topographical variables affected forest assembly more than soil variables. Species diversity was evaluated using the Shannon–Wiener, Simpson’s diversity, and Pielou’s evenness indexes. The environmental factors that affected species distribution had different effects on species diversity. The vegetation-environment relationship in the southern region was different than the central region of the Taihang Mountains, and vegetation restoration was at an early stage. The terrain of the southern region, especially elevation and slope, should be considered for vegetation restoration and conservation.

## Introduction

Forests in low mountains and hills are significant to forest conservation because they are rare and frequently disturbed by human activities^1,2^. The low mountains and hills in the southern region of the Taihang Mountains are located in northwest Henan Province. Deforestation of the southern Taihang Mountains for farming and lumber has a long history, with the discovery of oracle bone scripts (14^th^–11^th^ century B.C.), and is now the most populous province in China. Almost all the natural forest in the southern Taihang Mountains was clear-cut during the Daoguang period of the Qing Dynasty (i.e. the early 19^th^ century^3^). The entire Taihang Mountains forest coverage rate of 20–40% (8000 B.C.) was reduced to less than 5% (A.D. 1950). Compared with other areas of the Taihang Mountains, the forests in the southern hills were the most severely damaged^4^. Secondary forest was restored in the late 20^th^ century. The Macaca Nature Reserve was established in 1982 to protect the local *Macaca mulatta* population and forest ecosystem. In 1998, the Macaca National Nature Reserve was merged with the Taihangshan Macaques National Nature Reserve. Further, China implemented the natural forest protection project in 1998 and stopped the logging of all natural forests^5^. Zhang analysed the Landsat images of 1995–2000, and indicated that the forest coverage of Southern Taihang Mountains has recovered to 32%^6^. The relationship between community composition and environment, following long-term restoration of the natural secondary forest, is important to promote restoration of local vegetation, protect biological diversity, and understand the restoration process of the local ecosystem in this region.

The composition and structure of mountain vegetation is influenced by many environmental factors, including terrain, rainfall, geographic location, soil, and geological age^7^. The complexity of the terrain produces heterogeneity in light, water, and temperature, which drives species distribution, community differences, and landscape heterogeneity^8^. Soil factors also affect plant growth and soil nutrient levels (e.g. total N, available P, and available K) are positively correlated with plant growth^9^. Conversely, vegetation restoration improves soil properties like soil organic matter content, microbial activity, nutrient levels, and physical properties (e.g. bulk density, porosity, and water content), rather promoting nutrient cycling and plant growth^10^.

In order to explore vegetation restoration in the Taihang Mountains, studies have investigated plant community composition^11–13^ and plant species distribution^14^. Yang^15^ discussed the factors affecting forest growth in the Taihang Mountains. Zhang *et al*.^16,17^ evaluated the gradient of plant communities and Liu *et al*.^18^ analysed secondary forest-environment relationships over long-term natural restoration processes in the central Taihang Mountains. Previous studies have largely focused on the central and northern regions of the Taihang Mountains, but few reports are available regarding the secondary forest-environment relationships in the southern Taihang Mountains. The overall framework of quantitative understanding of vegetation restoration in the Taihang Mountains is incomplete.

The Taihang Mountains have a long north–south range of over 400 km and a 290 km east-west range, with high elevation in the north and low elevation in the south. They represent the convergence zone of the southern and northern floras of the East Asian continent, and species composition is very transitional. The difference in terrain, climate, and latitude produces significant variation in north-south vegetation composition^19^. Compared with the central and northern regions, the damage to vegetation in the southern region is more severe^4^, along with soil and water loss. The soil is barren, and the disturbance from human activities is frequent, which has made vegetation restoration difficult^6,18^. As such, we assumed that restoration in this area was still in the early stages, and the community composition and vegetation-environment relationship were different from that in the central and northern regions; therefore, the vegetation reconstruction and restoration processes used in the central and northern regions cannot be directly applied to this area. In this study, we investigated the natural secondary-deciduous broad-leaved forest community in the hills of the southern Taihang Mountains. We analysed the environmental gradient, and discussed the unique community composition and its relationship with the environment to provide a scientific basis for southern afforestation and protection projects.

This study had three aims: first, to comprehend the typical community composition following long-term natural recovery; second, to analyse the spatial heterogeneity of water, light, temperature, and nutrients and identify the key environmental variables; and third, to reveal the environmental gradients that affect community composition and species distribution, and comprehensively understand the mechanisms that create the spatial heterogeneity of natural secondary forest assemblies in this region.

## Results

### Descriptive statistical analysis and correlation analysis for the environmental data

The dispersion ranges and the central tendencies of elevation, slope, and all soil variables were estimated using the minimum-maximum and mean-median values (Table 1). The variability of environmental data was estimated using coefficient of variation CV values. Soil bulk density, soil total porosity, and pH had low CV values (<15%), while soil gravel content and organic C had high CV values (>65%).

**Table 1:** Descriptive statistical analysis of the topography and soil data.

Environmental variable	Minimum	Maximum	Mean	Median	CV
Elevation (m)	310	1252	815.1	827.5	34%
Slope (°)	21	70	35.4	35.5	29%
Soil bulk density (g·cm^−3^)	0.84	1.47	1.11	1.13	14%
Soil total porosity (% vol.)	44.6	68.2	58.2	57.4	10%
Soil moisture content (% weight)	4	27	13	12	50%
Soil electrical conductivity (dS·m^−1^)	1.41	9.14	3.94	3.29	56%
Soil pH (−log(H^+^))	4.95	7.90	6.51	6.52	12%
Soil gravel content (>2 mm, % vol.)	0	28	11.3	11.5	67%
Soil depth (cm)	8	20	13.4	13.5	32%
Soil total N (% weight)	0.21	1.28	0.61	0.55	48%
Soil organic C (% weight)	1.37	15.00	5.66	3.93	71%

The R^2^ of the correlation between the environmental variables is shown in Table 2. Elevation and soil total N showed a significant, positive correlation at the 0.01 level. Slope position and soil electrical conductivity showed a significant, positive correlation at the 0.01 level. Soil bulk density showed a significant, negative correlation with soil moisture content, electrical conductivity, total N, and organic C at the 0.01 level. Soil organic C also showed a significant, positive correlation with soil total porosity, electrical conductivity, and total N at the 0.01 level. There was a clear correlation between soil nutrient variables, and soil nutrients were closely related to soil bulk density and total porosity. There was no significant correlation between soil variables and slope or aspect.

**Table 2:** Correlation analysis of the environmental variables in the southern Taihang Mountains.

Environmental variable	Ele	Asp	Slo	SP	BD	TP	MC	EC	pH	GC	Dep	TN	OC
Ele	1												
Asp	0.158	1											
Slo	−0.131	−0.045	1										
SP	−0.074	0.051	0.247	1									
BD	−0.394	−0.231	−0.124	−0.209	1								
TP	0.387	0.236	0.124	0.211	−1.000**	1							
MC	0.257	0.157	−0.289	0.230	−0.577**	0.581**	1						
EC	−0.242	−0.134	0.284	0.527**	−0.553**	0.557**	0.396	1					
pH	−0.388	−0.260	0.280	0.155	−0.060	0.066	0.107	0.452*	1				
GC	0.061	−0.109	0.137	0.145	0.447*	−0.452*	−0.501*	−0.226	−0.067	1			
Dep	0.316	−0.044	−0.276	0.260	0.011	−0.016	0.282	−0.226	0.066	0.120	1		
TN	0.564**	0.019	0.052	0.238	−0.636**	0.632**	0.434*	0.423*	0.020	0.151	0.046	1	
OC	0.416*	−0.072	0.082	0.327	−0.659**	0.657**	0.504*	0.570**	0.146	0.133	0.018	0.957**	1

Asp, slope aspect; Slo, slope; SP, slope position; BD, soil bulk density; TP, soil total porosity; MC, soil moisture content; EC, soil electrical conductivity; pH, soil pH (H_2_O); GC, soil gravel content; Dep, soil depth; TN, soil total N; OC, soil organic C; *significance at the 0.05 level (2-tailed); **significance at the 0.01 level (2-tailed).

### Two-way indicator hydrocarbon analysis

**Figure 1:**
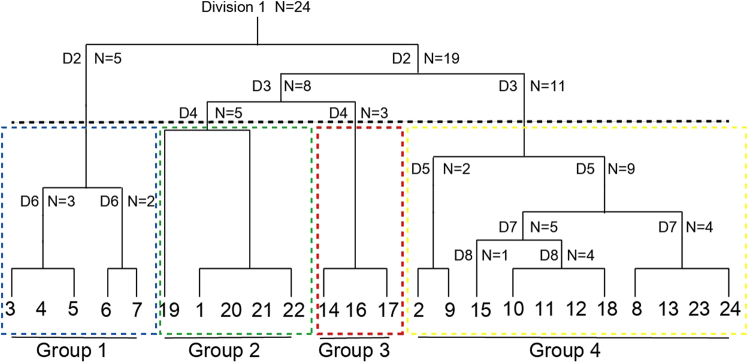
TWINSPAN dendrogram for 24 quadrats.

Two-way indicator hydrocarbon analysis (TWINSPAN) classified the 24 samples into four groups representing four forest types (Fig. 1). The names and habitats are described below.

Group 1. *Gleditsia microphylla*-*Quercus baronii* forest type. Distributed from 300 to 800 m on mid and lower slopes of hills. Disturbance intensity is heavy to very heavy. The soil depth is 10–15 cm with mildly alkaline soil (pH 7.5–8.0). Both the soil gravel content (0.5–9.5%) and soil total N (0.2–0.8%) are low. The community has a total cover of 90%, with absolute tree, shrub, and herb cover of 40–70%, 30–70%, and 10–40%, respectively. The common species in the tree layer are *Gleditsia microphylla*, *Quercus baronii*, *Cotinus coggygria*, *Prunus davidiana*, and *Fraxinus bungeana*. The common species in the shrub layer are *Vitex negundo* and *Gleditsia microphylla*.

Group 2. *Quercus variabilis*-*Koelreuteria paniculata* forest type. Distributed from 300 to 700 m in the hills. Disturbance intensity is moderate. The community has a total cover of 50–85%, with absolute tree, shrub, and herb cover of 50–80%, 20–40%, and 10–30%, respectively. The common species in the tree layer are *Quercus variabilis* and *Koelreuteria paniculata*. The common species in the shrub layer are *Vitex negundo*, *Rosa xanthine*, and *Spiraea trilobata*.

Group 3. *Acer mono* forest type. Distributed from 1000 to 1300 m in the low mountains. Disturbance intensity is light. The community has a total cover of 70–85%, with absolute tree, shrub, and herb cover of 70%, 25%, and 10–20%, respectively. The common species in the tree layer are *Acer mono, Cornus alba, Morus alba*, and *Acer grosser*. The common species in the shrub layer are *Forsythia suspense* and *Spiraea salicifolia*.

Group 4. *Cotinus coggygria*-*Quercus baronii* forest type. Distributed from 600 to 1300 m the hills and low mountains. Disturbance intensity is light and moderate. The community has a total cover of 60–90%, with absolute tree, shrub, and herb cover of 20–70%, 10–70%, and 10–60%, respectively. The common species in the tree layer are *Quercus baronii*, *Cotinus coggygria*, and *Forsythia suspense*. The common species in the shrub layer are *Vitex negundo*, *Cotoneaster multiflorus*, *Cotinus coggygria*, and *Forsythia suspense*.

### Detrended Correspondence Analysis and Corresponding Canonical Ordination Analysis

The maximum value of the detrended correspondence analysis (DCA) (Supplementary Table S1) axis lengths was 7.6693 > 4. The variation in species distribution showed a mostly unimodal relationship with environmental variables^9,20^. Therefore, we used the corresponding canonical ordination analysis (CCA) to analyse the relationship between species and environmental variables.

The CCA (Table 3) showed the overall variance was partitioned into constrained (68.17%) and unconstrained (31.83%) fractions. The constrained fraction was the amount of variance in species matrix explained by the environmental variables. The CCA yielded 13 constrained axes and 10 unconstrained axes for the residuals. The cumulative proportional value of the first two axes was 21.81%.

**Table 3:** Corresponding Canonical Ordination Analysis (CCA) and most parsimonious CCA (PCCA).

Axis	CCA			Parsimonious CCA		
	Eigenvalue	Proportion Explained	Cumulative Proportion	Eigenvalue	Proportion Explained	Cumulative Proportion
CCA1	0.77770	0.11490	0.11490	0.71510	0.10560	0.10560
CCA2	0.69920	0.10330	0.21810	0.64433	0.09517	0.20080
CCA3	0.65209	0.09632	0.31446	0.50601	0.07474	0.27554
CCA4	0.59446	0.08781	0.40226	0.47563	0.07025	0.34579
CCA5	0.48990	0.07236	0.47462	0.32289	0.04769	0.39349
CCA6	0.39209	0.05791	0.53254	0.21913	0.03237	0.42585
Total CCA axes	4.615	0.6817		2.883	0.4259	
CA1	0.46845	0.06919	0.06919	0.66099	0.09763	0.09763
CA2	0.39377	0.05816	0.12735	0.57395	0.08477	0.18240
CA3	0.35501	0.05244	0.17979	0.49214	0.07269	0.25509
CA4	0.29606	0.04373	0.22352	0.45298	0.06691	0.32200
CA5	0.25032	0.03697	0.26049	0.38699	0.05716	0.37916
CA6	0.12485	0.01844	0.27893	0.32687	0.04828	0.42744
Total CA axes	2.155	0.3183		3.887	0.5741	

CCA1–CCA6, the constrained axes 1–6 of CCA and PCCA; CA1–CA6, the unconstrained axes 1–6 of CCA and PCCA.

### Forward selection and most parsimonious CCA analysis

**Figure 2:**
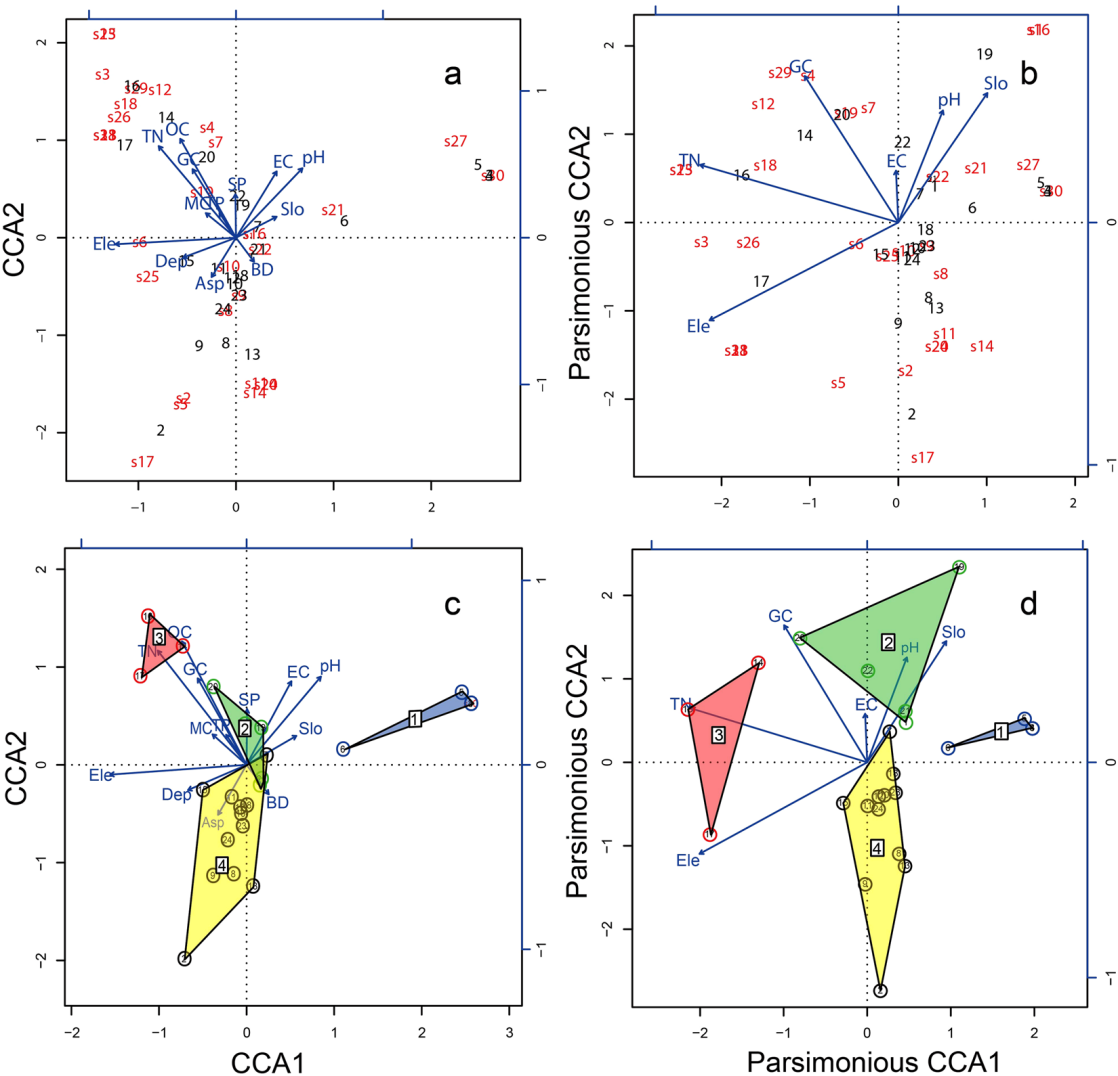
Corresponding Canonical Ordination Analysis (CCA) and Parsimonious CCA ordination plots with TWINSPAN result. (**a**) CCA triplot. (**b**) Parsimonious CCA biplot with four forest types. (**c**) CCA triplot. (**d**) Parsimonious CCA biplot with four forest types 2. The bottom and left-hand scales represent the quadrats and species, respectively. The top and right-hand scales represent the environmental variables. s1–s31 indicate tree species codes (see Supplementary Table S2); 1–24 indicate 24 quadrats. The blue, arrowed lines indicate environmental variables. Ele, elevation; Asp, aspect; Slo, slope; SP, slope position; BD, soil bulk density; TP, soil total porosity; MC, soil moisture content; EC, soil electrical conductivity; GC, soil gravel content; Dep, soil depth; TN, soil total N; OC, soil organic C.

Forward selection was conducted on the 13 environmental variables in turn until there was no obvious explanatory variable. This yielded six reserved environmental factors (elevation, soil total N, soil pH, soil electrical conductivity, soil gravel content, and slope). These six variables were used to re-run the CCA to obtain the most parsimonious CCA (PCCA). From Table 3, the overall variance in PCCA was partitioned into constrained (42.59%) and unconstrained (57.41%) fractions. Compared with CCA, the total number of explanatory variables in PCCA decreased by more than half, and the explained variation was only reduced by 25.58%. The variance inflation factors (VIFs) in the CCA indicated that soil bulk density (3,232.2), soil total porosity (3,268.0), soil total N (43.1), and soil organic C (84.3) had strong collinearity in the CCA (Supplementary Table S2). In order to reduce the number of explanatory variables and avoid strong collinearity among variables, we used forward selection to model the most parsimonious CCA: the explanatory variable that has the most significant partial effect is selected one by one, until no more significant variable can enter the model. Linear dependencies can be explored by computing the selected variables’ VIF, which measures how much a regression coefficient is inflated by the presence of other explanatory variables^21^. VIFs above 20 indicated strong collinearity. Ideally, VIFs above 10 should be at least examined, and avoided if possible^22^. In this study, the PCCA model of secondary forest had no harmful collinearity (VIFs of 6 variables were below 3.5, see Table 4) and was highly significant. Moreover, the distribution discrepancy of the four forest types (groups 1–4) was clearer in the PCCA biplot (Fig. 2d) than in the CCA triplot (Fig. 2c). Therefore, forward selection was effective.

**Table 4:** Permutation test for the environmental variables of parsimonious CCA.

Environmental variable	Chi square	F value	Pr(>F)
Elevation	0.6531	2.8561	0.001^***^
Soil total N	0.5248	2.2951	0.001^***^
Soil gravel content	0.4535	1.9832	0.001^***^
Slope	0.4993	2.1838	0.004^**^
Soil electric conductivity	0.4001	1.7500	0.033^*^
Soil pH	0.3524	1.5410	0.062.

***Significance at the 0.001 level; **significance at the 0.01 level; *significance at the 0.05 level; ^.^Correlation significance at the 0.1 level.

Figure 2b shows that *Euptelea pleiospermum* (s13), *Zelkova sinica* (s28), and *Acer davidii* (s31) were mostly distributed in higher mountain areas. The habitats of *Ailanthus altissima* (s1) and *Pteroceltis tatarinowii* (s16) were on higher slopes with good drainage. *Broussonetia papyrifera* (s4) and *Celtis bungeana* (s29) favoured soil with more gravel content, and *Toxicodendron vernicifluum* (s15) and *Ulmus lamellosa* (s23) favoured more soil N content and habitats with sufficient nutrients.

### Permutation tests for environmental factors

The results of permutation tests for the effects of environmental variables on the PCCA model are given in Table 4. Forest assembly was mostly affected by elevation, soil total N, and soil gravel content (0.001 level), and then (in order) by slope, soil electrical conductivity, and pH. Permutation tests for the significance of each environmental variable on the canonical axes of the CCA and PCCA are given in Supplementary Table S4. Elevation and canonical axes of the CCA and PCCA were significantly correlated at the 0.001 level (R^2^ = 0.6418, 0.7285). Both soil total N and soil organic C were significantly correlated with the canonical axes of the CCA at the 0.01 level (R^2^ = 0.6499, 0.5870) and the canonical axes of the PCCA at the 0.001 level (R^2^ = 0.6543, 0.5354). Supplementary Table S4 shows that the soil total N and elevation played an important role in PCCA axis 1; therefore, PCCA axis 1 represented nutrient and elevation gradients. The soil gravel content explained a higher proportion of variance in PCCA axis 2, which represented the physical properties of soil. From left to right along PCCA axis 1 (Fig. 2d), the trend of forest-type distribution was from group 3 to groups 2 and 4, and then to group 1. From the bottom to the top along PCCA axis 2, the trend of forest-type distribution was from group 4 to groups 1 and 3, and then to group 2.

### Species abundance and diversity analyses

**Figure 3:**
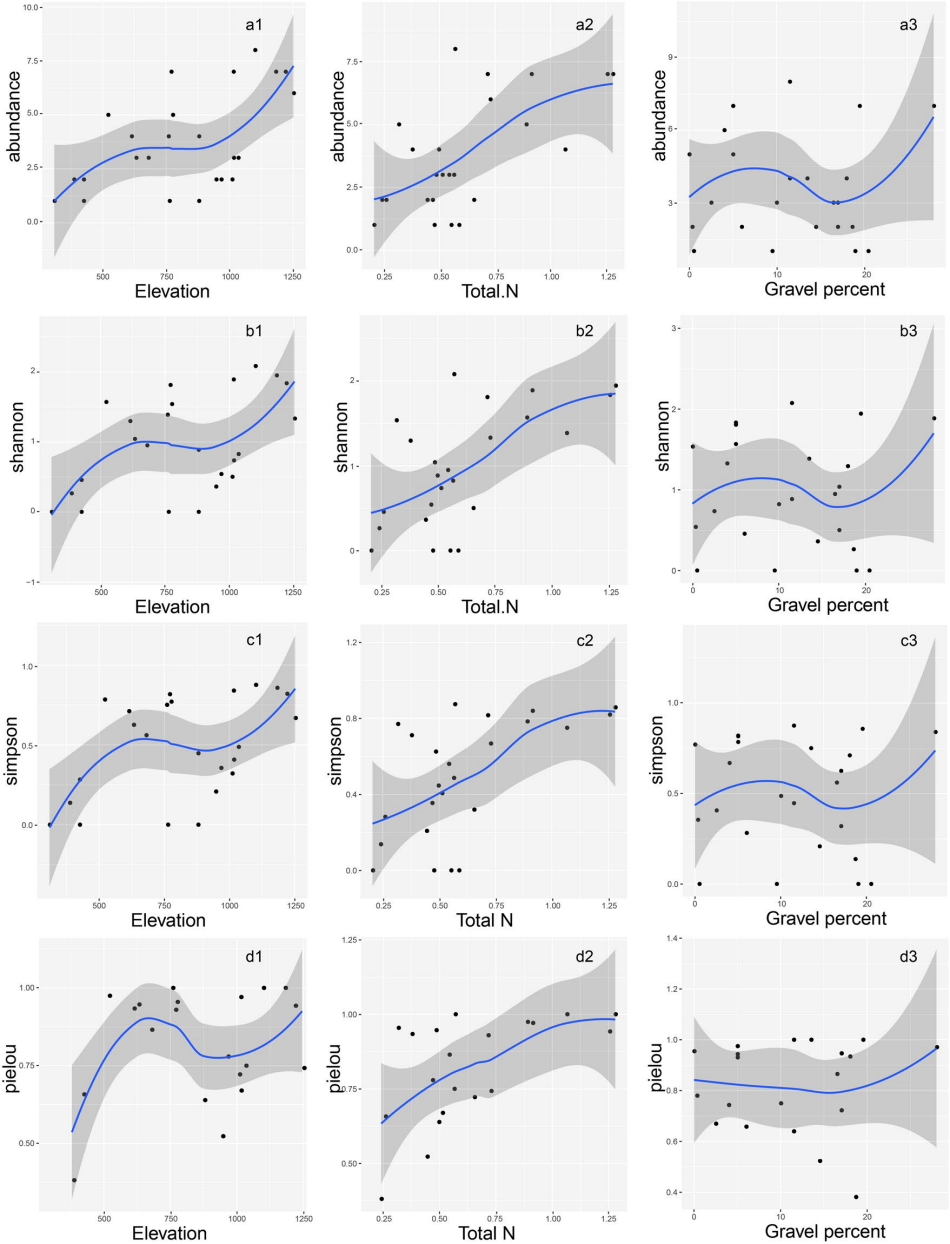
The abundance (**a**1–**a**3), Shannon (**b**1–**b**3), Simpson (**c**1–**c**3), and Pielou (**c**1–**c**3) indexes of 24 quadrats on the elevation, soil total N, and soil gravel percent.

The species abundance, Shannon–Wiener’s, Simpson’s predominance, and Pielou’s evenness indexes are shown in Supplementary Table S5. On the basis of the result of permutation test for environmental variables, we chose the three most significant variables for the community assembly, namely, elevation, soil total N, and soil gravel content (significance at the 0.001 level, Table 4). A scatter plot (Fig. 3) was created to explore changes in species abundance and diversity indexes according to the changes in these three variables. Interestingly, the environmental factors that affected species distribution had different effects on species diversity. The species abundance and diversity indexes increased with increase in elevation. However, this trend in increase was more pronounced in areas with an elevation of less than 500 m and greater than 1000 m. Species abundance and diversity indexes showed a steady upward trend with increase in the soil total N, but a slow increase when the total N was more than 0.75%. All the indexes showed no obvious change in trend with variations in the soil gravel content.

## Discussion

We assessed the relationship between multiple environmental variables and community assembly of natural secondary forests in the southern Taihang Mountains. Thirteen terrain and soil variables jointly explained 68.17% of the total variance. The results showed that the composition of the natural secondary forest in the southern Taihang Mountains was closely related to topographical features and soil properties. Among the terrain factors, elevation was the most significant, followed by slope. The soil factors that most influenced community assembly were (in order) soil total N, organic C, pH, and gravel content.

There were strong correlations between all environmental variables in the study area. Soil organic C, soil total porosity, electrical conductivity, and total N showed significant, positive correlations. Previous studies have shown a significant, positive correlation between plant growth and soil nutrients; vegetation restoration increased soil organic matter content and improved soil properties^9^. The increase in soil organic matter also significantly affected microbial activity, soil nutrients, and physical properties, which in turn promoted nutrient cycling and plant growth^10^. The significant correlation between soil variables reflected the nutrient and material cycling in secondary forest ecosystems in the study area. Furthermore, there was a significant, positive correlation between soil nutrients (soil total N, organic C) and elevation. Unlike the soil variables, the terrain variables were not directly measured, as they were composite representations of light, water, temperature, and soil properties^23^.

In the high-altitude mountainous area, increasing elevation produces an adiabatic decline in temperature. Declining temperature has an overarching influence on vegetation responses, soil microbial properties, and nutrients^24^. However, the study area was located in the low mountains and hills adjoining the populated plains. The lower the altitude, the more disturbed the vegetation, which reduced soil organic matter and nutrient levels. This may explain the significant, positive correlation between elevation and soil nutrients; high altitude limits the human activity that disturbs the secondary forest at lower elevations. Soil conductivity was significantly, positively correlated with slope position and pH. Soil conductivity increased from uphill to downhill, and the soil tended to be alkaline. Since the soil parent material in the Taihang Mountains is limestone, the overall soil was meta-alkalescence. The soluble salts in the upper slope soils gradually converge in the bottom soil via leaching, and then rise to the surface as part of the evaporation process. Consequently, the bottom soil had a higher pH and soil conductivity. The alkaline soil, along with better water conditions, was conducive to soil microbial activity, which ultimately facilitated nutrient and material cycling.

The strong correlations among the environmental variables in the study area could produce unstable regression coefficients of the explanatory variables in the model^9^. In order to reduce the number of explanatory variables and avoid strong collinearity among variables, we used forward selection to model the most parsimonious CCA. The PCCA model of secondary forest had no harmful collinearity and showed the effects of key environmental variables clearly. The PCCA model was composed of two terrain variables (elevation and slope) and four soil variables (total N, gravel content, electrical conductivity, and pH). The effect of elevation on community assembly was the highest, followed by soil nutrients, represented by soil total N, and soil physical properties, represented by soil gravel content. This was different from the results by Zhang *et al*.^17^ and Liu *et al*.^18^ in the central Taihang Mountains, which reported an effect mostly due to soil nutrients. The effect of elevation was less than slope and aspect^17,18^. This may have been due to the smaller decrease in elevation and better vegetation conditions in the central Taihang Mountains. Wang *et al*. argued that the difference in elevation between the quadrats determined the extent of influence of elevation^9^. More precisely, where the difference value was less than 300 m, the effect of elevation gradient on species distribution was very slight^9^. This can explain why elevation plays an important role in the southern and northern regions, but is less influential in the central region. The effect of environmental variables in the southern region was similar to that in the northern region. Zhang *et al*.^25^ found the critical influential variable in the northern region was elevation, followed by soil nutrients. Overall, the effects of environmental variables were similar in the southern and northern regions, but different in the central region of the Taihang Mountains. The dominant species of secondary forest in the middle of the Taihang Mountains were the same as that in the northern region, including *Quercus variabilis*, *Quercus aliena*, *Cotinus coggygria*, and *Carpinus turczaninovwii* ^17,26^. *Betula platyphylla*, *Larix principis-rupprechtii*, and *Quercus liaotungensis* were commonly noted in the forest community of the middle region. In the community of the northern region, the dominance of *Carpinus turczaninowii*, *Quercus variabilis*, and *Quercus aliena* was found to decrease, and that of *Quercus liaotungensis*, *Larix principis-rupprechtii*, and *Pinus tabulaeformis* increased^27^. Thus, the Taihang Mountains, which represent the convergence zone of the southern and northern floras of the East Asian continent, showed a tendency of species turnover from deciduous broadleaf to coniferous species along with increase in elevation and dimension from the south to north.

In contrast to the original forest community, the secondary forest was relatively heterogeneous^28^. Our study showed that the community composition of the secondary forest in the southern Taihang Mountains was notably heterogeneous along an environmental gradient. Twenty-four samples were classified into four forest types (groups 1–4). The PCCA enabled ordination of the four forest types more distinctly than the CCA. These forest types were dispersed along the main ecological gradient, represented by PCCA1 and PCCA2. The forest types changed from group 3 to groups 2 and 4, and then to group 1 from left to right of PCCA1. PCCA1 represented the variance in elevation and soil nutrients. As the elevation decreased, the soil nutrients reduced from left to right of PCCA1. This indicated that forest type1 and type 3 had obvious preferences for soil nutrients and different altitude gradients. As the dominant species of type 3, *Acer mono* is suitable for growing in high-altitude areas with fertile soil. *Gleditsia microphylla* and *Quercus baronii*, the dominant species in forest type 1, are able to growth and reproduce in low-altitude areas with poor soil. These two species can be used as constructive species in local vegetation restoration. PCCA2, which represented variance in soil pH and water conditions, was related to slope, soil gravel content, and pH. High slope and soil gravel content imply good drainage, which was also shown in the positive correlation of soil water content. Soil gravel content increased, slope increased, soil pH increased, and the water content decreased from the bottom to top of PCCA2.The forest types changed from group 4 to groups 1 and 3, and then to group 2. The differences in spatial distribution between forest type 2 and type 4 reflected the difference in preference for water conditions. *Quercus variabilis* and *Koelreuteria paniculata*, the dominant species in forest type 2, were distributed along steep slopes with good drainage, and adapted to alkali soil. *Cotinus coggygria* and *Quercus baronii*, the dominant species in forest type 4, preferred good moisture content and acidic soil with low gravel content. Consequently, the spatial difference in nutrients and water over different terrain were the main environmental factors affecting tree species distribution and community assembly of natural secondary forest in the southern Taihang Mountains. That is, the terrain indirectly affected the distribution of tree species and community assembly by affecting soil properties, and restricting human disturbance, forming the second-order drivers. Our research showed that elevation significantly affected *Gleditsia microphylla*, *Quercus baronii*, *Gleditsia microphylla*, and *Quercus baronii* communities. Slope significantly affected *Quercus variabilis*, *Koelreuteria paniculata*, *Cotinus coggygria*, and *Quercus baronii* communities.

The environmental variables also affected species abundance and diversity in the north Taihang Mountains. Elevation, soil total N, and soil gravel content were the 3 most significant environmental variables for species distribution and community assembly, but the effects of these 3 variables on species abundance and diversity were varied. Species abundance and diversity increased with increase in elevation and was obviously low in regions of low elevation (<500 m). This indicated that human disturbance seriously affects species abundance and diversity of secondary forests. Our study showed that tree species abundance, Shannon–Wiener’s, and Simpson’s indexes in the southern region increased with increase in elevation, similar to the results of Li^26^ in the middle region (elevation 1050–2089 m). However, Pielou’s index, which reflects species evenness, decreased with increase in elevation. As dimension and elevation increase from the southern to middle regions of Taihang Mountains, the broad-leaved forest was observed to be gradually replaced by coniferous forest, with reduced species evenness. Species abundance and diversity indexes showed a steady upward trend with increase in soil total N. This indicated a positive correlation between species diversity and the soil nutrient condition of secondary forest. Although the community assembly and species distribution were significantly affected by soil gravel content, no obvious effect was noted on species abundance and diversity. This indicated that local species have a different selection strategy on ecological niche, and the existing tree species can adapt well to different levels of soil gravel content. As the original forest in the low elevation region was clear cut in the study area, there are very few original forests at the peak of elevation >1200 m, where the vegetation types are mixed broadleaf–conifer forest. In order to explore the existing secondary forest community that has been less disturbed, we investigated 7 quadrats that were located far away from the city (1000 m < elevation < 1220 m). These 7 quadrats aggregated in forest type 3 and partly type 4, which was in clear contrast to the forest type 1 found close to the city and facing major disturbances. Forest types 3 and 4 provide a model for afforestation and protection projects in southern hilly areas and low mountains. In the early stage of vegetation restoration, community assembly is more correlated with light, water, and temperature. As restoration proceeds, community assembly gradually becomes more correlated with soil nutrients^29^. This study in the southern Taihang Mountains showed that elevation was a key factor affecting the community and tree species. The soil total N, soil electrical conductivity, and soil moisture content were also significantly affected by elevation, slope, and slope position. Therefore, the differences in water, nutrients, and human disturbance with terrain were the main factors in secondary forest assembly in the southern Taihang Mountains. We argue that forest restoration in the southern Taihang Mountains is at an early stage compared to that in the central region. The hills and low mountains in the southern region are the current focus of vegetation restoration and conservation in the Taihang Mountains. In the southern region, vegetation restoration should take full account of the influence and restriction of terrain factors, especially elevation and slope. We suggest employing the four forest types in afforestation programs in the southern Taihang Mountains: type 1, *Gleditsia microphylla*-*Quercus baronii*, for low altitudes; type 2, *Quercus variabilis*-*Koelreuteria paniculata*, for mid to upper slopes with good drainage; type 3, *Acer mono*, for high altitudes with good nutrients; and type 4*, Cotinus coggygria*-*Quercus baronii*, for bottom to lower slopes with good water and soil conditions.

## Conclusions

The composition of natural secondary forest in the southern Taihang Mountains was closely related to the topographical features and soil properties. The secondary forest assembly was influenced by spatial differences in water and nutrients driven by terrain. The terrain indirectly influenced community composition and tree species distribution by influencing soil properties and restricting human disturbance, forming second-order drivers. Six main environmental variables identified in the PCCA model were elevation, slope, soil total N, gravel content, soil electrical conductivity, and pH. Elevation played the most important role in forest assembly, followed by soil nutrients, represented by soil total N, and soil physical properties, represented by soil gravel content. The effects of environmental variables were similar in the southern and northern regions, but different in the central region.

Twenty-four samples of natural secondary forest were classified into four groups. *Gleditsia microphylla*-*Quercus baronii* forest type (group 1) and *Acer mono* forest type (group 3) had obvious preferences for soil nutrients and differences in elevational gradient. The difference in spatial distribution between *Quercus variabilis*-*Koelreuteria paniculata* forest type (group 2) and *Cotinus coggygria*-*Quercus baronii* forest type (group 4) reflected the difference in preferences for water conditions. Our results differed from similar studies in other regions of the Taihang Mountains, demonstrating the need to account for small- and large-scale variation to ensure the success of afforestation and conservation projects across a region. Forest restoration in the southern Taihang Mountains is at early stage compared to the central region. The hills and low mountains in the southern region are the current focus of afforestation and conservation in the Taihang Mountains. The terrain factors of the southern region, especially elevation and slope, should be considered for vegetation restoration and conservation.

## Methods

### Study area

This study was conducted in northwest Henan Province (34°53′–35°16′N, 112°01′–112°45′E), China. The climate of the study area was warm temperate and semi-humid with continental characteristics. The average annual mean temperature was 14.5 °C. The annual precipitation varied from 440 to 860 mm. The average annual precipitation was 568 mm, which mainly occurred from July to September^30,31^. The zonal soil type was limestone soil and some was shale soil. The mean soil depth was 20 cm. The soil characteristics were slow weathering, strong water leakage, and thin, non-stratified soil layers^32^.

### Quadrat survey and sampling

Based on general investigation^9,16^, 24 quadrats (20 m × 20 m) of natural secondary forest were sampled from August to October 2015. All trees with diameter at breast height ≥1.0 cm and height ≥1.5 m were identified and measured. The scientific names of tree species were in accordance with the *Chinese Biological Species Directory* ^33^. A total of 31 tree species in 24 quadrats was recorded (Supplementary Table S3).

Three to four soil sub-samples per quadrat were collected, using a soil corer of 10 cm × 10 cm. The soil sub-samples were pooled into one composite sample per quadrat. The composite samples were dried, thoroughly mixed, and passed through a 2-mm sieve to separate organics, soil, and gravel. Nine soil variables were measured, including soil depth, soil bulk density, soil organic C, soil pH (H_2_O), soil total N, soil gravel content, soil total porosity, soil moisture content, and soil electrical conductivity. Two replicates were averaged for each sample to improve precision.

Four topographical factors, including elevation, slope, slope aspect, and slope position of each quadrat were measured. Elevation was measured using a portable GPS (eTrex10). We measured the slope and aspect in each quadrat by using an electronic total station (DTM-102N). Slope position and aspect were converted into a coded scale using membership functions and an empirical formula based on Wang^9^, Liu^34^ and Liu^35^ studies. Slope position was classified as 0.4 for upper slope, 1.0 for middle slope, and 0.8 for lower slope. Slope aspect was classified as 0.3 for a sunny slope, 0.5 for a half-sunny slope, 0.8 for a half-shaded slope, and 1.0 for a shaded slope^9^. The datasets and analyses from the current study are available from the corresponding author upon request.

### Statistical analysis

Descriptive statistics, including the mean, median, CV, and minimum and maximum values, were calculated for all environmental variables, except slope position and aspect. Pairwise correlations were used to analyse the relationships between environmental variables. The Importance Value (IV) was used in a multivariate analysis of forests and species diversity^36,37^. The IV of tree species was calculated using equation (1)^38^.

1 $${\rm{IV}}=({\rm{Relative}}\,{\rm{dominance}}+{\rm{Relative}}\,{\rm{frequency}}+{\rm{Relative}}\,{\rm{density}})/3$$

where, relative dominance refers to the sum of basal areas of a tree species within a quadrat, relative frequency refers to the percentage of quadrats containing a species over the total number of quadrats, and relative density refers to the number of tree species within a quadrat per unit area.

TWINSPAN analysis was conducted to cluster the 24 samples into different forest types. CCA was used to explore the relationships between forest assembly and environmental factors. We used the length of the DCA axis to identify the fit (linear or unimodal) of the model to species distribution^39^. The triplot and biplot that overlapped with the TWINSPAN results were used to analyse the relationship between tree species, environmental factors, quadrats, and forest types. Forward selection was used to compute the most parsimonious model based on significant explanatory variables. Species diversity was evaluated using the Shannon–Wiener, Simpson’s diversity, and Pielou’s evenness indexes^14,39^. The VIF of variables was used to measure how much a regression coefficient is inflated by the presence of other explanatory variables^21^. All analyses were conducted using R version 3.4.0 with “vegan” and “twinspanR” packages. Finally, we used the Monte Carlo 1000 permutations test to assess the significance of the F-values of environmental variables in the PCCA on the response variables (tree species and quadrats), and to test the significance of the R^2^ of each environmental variable in the canonical axes of CCA and PCCA^9^.

## Electronic supplementary material

Supplementary Tables S1-S5
