# Supplementary material for: The Relationship between Secondary Forest and Environmental Factors in the Southern Taihang Mountains
Source: Sci Rep. 2017 Nov 27;7:16431. doi: 10.1038/s41598-017-16647-0 (PMC5703897; doi:10.1038/s41598-017-16647-0)
Supplement: Supplementary file 1 — Supplementary Tables S1-S5 [file 41598_2017_16647_MOESM1_ESM.doc]

**The Relationship between Secondary Forest and Environmental Factors in the Southern Taihang Mountains**

Hui Zhao^1,2^, Qi-Rui Wang^2^, Fan Wei^2^, Guo-Hua Song^1,3,*^

| **Axis** | **Eigenvalue** | **Decorana value** | **Axis length** |
| --- | --- | --- | --- |
| DCA1 | 0.8605 | 0.8751 | 7.6693 |
| DCA2 | 0.5920 | 0.6271 | 4.4482 |
| DCA3 | 0.5637 | 0.3797 | 4.3571 |
| DCA4 | 0.2960 | 0.2493 | 2.2300 |

**Supplementary Table S1. Results of the first four axes of DCA.**

| **Environmental variable** | **VIF in CCA** | **VIF in PCCA** |
| --- | --- | --- |
| Elevation | 3.194588 | 2.481378 |
| Slope aspect | 3.314994 |  |
| Slope | 2.040789 | 1.25113 |
| Slope position | 2.515052 |  |
| Soil bulk density | 3232.20475 |  |
| Soil total porosity | 3268.04256 |  |
| Soil moisture content | 7.600079 |  |
| Soil electric conductivity | 5.973132 | 2.925148 |
| Soil pH | 2.385554 | 1.415482 |
| Soil gravel content | 9.138676 | 1.369105 |
| Soil depth | 3.998632 |  |
| Soil total N | 43.077709 | 3.467808 |
| Soil organic C | 84.304713 |  |

**Supplementary Table S2. Variance inflation factors (VIF) of the environmental variables.**

| **Specie** | **Code** |
| --- | --- |
| *Ailanthus altissima* | s1 |
| *Carpinus turczaninowii* | s2 |
| *Acer grosseri* | s3 |
| *Broussonetia papyrifera* | s4 |
| *Malus honanensis* | s5 |
| *Quercus aliena* | s6 |
| *Koelreuteria paniculata* | s7 |
| *Pistacia chinensis* | s8 |
| *Cotinus coggygria* | s9 |
| *Quercus baronii* | s10 |
| *Forsythia suspensa* | s11 |
| *Swida macrophylla* | s12 |
| *Euptelea pleiospermum* | s13 |
| *Abelia biflora* | s14 |
| *Toxicodendron vernicifluum* | s15 |
| *Pteroceltis tatarinowii* | s16 |
| *Quercus aliena var. acuteserrata* | s17 |
| *Morus alba* | s18 |
| *Juglans cathayensis* | s19 |
| *Morus mongolica var. diabolica* | s20 |
| *Prunus davidiana* | s21 |
| *Quercus variabilis* | s22 |
| *Ulmus lamellosa* | s23 |
| *Kolkwitzia amabilis* | s24 |
| *Evodia daniellii* | s25 |
| *Acer truncatum* | s26 |
| *Fraxinus bungeana DC.* | s27 |
| *Zelkova Sinica* | s28 |
| *Celtis bungeana* | s29 |
| *Gleditsia microphylla* | s30 |
| *Acer davidii* | s31 |

**Supplementary Table S3. Codes of 31 tree species.**

| **Environmental variable** | **CCA1** | **CCA2** | **R^2^** | **Pr(>r)** | **PCCA1** | **PCCA2** | **R^2^** | **Pr(>r)** |
| --- | --- | --- | --- | --- | --- | --- | --- | --- |
| Elevation | -0.99776 | -0.06692 | 0.6418 | 0.000999^***^ | -0.88377 | -0.46792 | 0.7285 | 0.000999^***^ |
| Slope aspect | -0.53691 | -0.84364 | 0.1013 | 0.372627 | -0.19728 | -0.98035 | 0.0356 | 0.725275 |
| Slope | 0.88513 | 0.46535 | 0.1106 | 0.333666 | 0.58274 | 0.81266 | 0.4072 | 0.010989^*^ |
| Slope potition | 0.01390 | 0.9999 | 0.0903 | 0.413586 | -0.22533 | 0.97428 | 0.1194 | 0.354645 |
| Soil bulk density | 0.64206 | -0.76665 | 0.0404 | 0.663337 | 0.91586 | 0.40151 | 0.1022 | 0.382617 |
| Soil total porosity | -0.60215 | 0.79838 | 0.0391 | 0.676324 | -0.91462 | -0.40431 | 0.0981 | 0.406593 |
| Soil moisture content | -0.77964 | 0.62622 | 0.0651 | 0.519481 | -0.76891 | -0.63936 | 0.2098 | 0.102897 |
| Soil electric conductivity | 0.51321 | 0.85826 | 0.2756 | 0.054945 | -0.01505 | 0.99989 | 0.0434 | 0.673327 |
| Soil pH | 0.68401 | 0.72947 | 0.4211 | 0.004995^**^ | 0.39647 | 0.91805 | 0.2405 | 0.078921 |
| Soil gravel content | -0.53113 | 0.84729 | 0.3020 | 0.035964^*^ | -0.53388 | 0.84556 | 0.4720 | 0.001998^**^ |
| Soil depth | -0.93182 | -0.36293 | 0.1359 | 0.264735 | -0.86231 | -0.50638 | 0.0926 | 0.444555 |
| Soil total N | -0.65373 | 0.75673 | 0.6499 | 0.001998^**^ | -0.96808 | 0.25063 | 0.6543 | 0.000999^***^ |
| Soil organic C | -0.50210 | 0.86481 | 0.5870 | 0.001998^**^ | -0.94016 | 0.34072 | 0.5354 | 0.000999^***^ |

**Supplementary Table S4. Permutation test for environmental factors to the axes of CCA and parsimonious CCA.** ‘***’ significance at the 0.001 level; ‘**’ significance at the 0.01 level; ‘*’ significance at the 0.05 level.

| **Plot** | **Abundance** | **Shannon** | **Simpson** | **Pielou** | **Elevation** (m) | **Gravel percent** (>2mm, % vol.) | **Total N**  (% weight) |
| --- | --- | --- | --- | --- | --- | --- | --- |
| p1 | 2 | 0.264 | 0.137 | 0.381 | 387 | 18.7 | 0.240 |
| p2 | 2 | 0.540 | 0.355 | 0.780 | 967 | 0.33 | 0.468 |
| p3 | 1 | 0 | 0 | NA | 310 | 0.5 | 0.476 |
| p4 | 1 | 0 | 0 | NA | 425 | 9.5 | 0.205 |
| p5 | 2 | 0.456 | 0.282 | 0.657 | 425 | 6.0 | 0.259 |
| p6 | 5 | 1.568 | 0.784 | 0.975 | 521 | 5.0 | 0.890 |
| p7 | 7 | 1.810 | 0.816 | 0.930 | 770 | 5.0 | 0.715 |
| p8 | 5 | 1.536 | 0.770 | 0.955 | 776 | 0 | 0.316 |
| p9 | 6 | 1.330 | 0.668 | 0.742 | 1252 | 4.0 | 0.728 |
| p10 | 2 | 0.362 | 0.208 | 0.523 | 947 | 14.5 | 0.444 |
| p11 | 3 | 0.736 | 0.406 | 0.670 | 1016 | 2.5 | 0.512 |
| p12 | 2 | 0.500 | 0.320 | 0.722 | 1010 | 17.0 | 0.654 |
| p13 | 3 | 0.823 | 0.486 | 0.750 | 1034 | 10.0 | 0.564 |
| p14 | 7 | 1.889 | 0.840 | 0.971 | 1015 | 28.0 | 0.913 |
| p15 | 8 | 2.079 | 0.875 | 1 | 1100 | 11.5 | 0.569 |
| p16 | 7 | 1.95 | 0.857 | 1 | 1182 | 19.5 | 1.280 |
| p17 | 7 | 1.834 | 0.820 | 0.942 | 1220 | 5.0 | 1.255 |
| p18 | 4 | 1.295 | 0.711 | 0.933 | 614 | 18.0 | 0.377 |
| p19 | 3 | 0.950 | 0.560 | 0.865 | 680 | 16.5 | 0.542 |
| p20 | 4 | 1.386 | 0.750 | 1 | 759 | 13.5 | 1.065 |
| p21 | 1 | 0 | 0 | NA | 763 | 20.5 | 0.587 |
| p22 | 3 | 1.040 | 0.625 | 0.946 | 632 | 17.0 | 0.484 |
| p23 | 1 | 0 | 0 | NA | 879 | 19.0 | 0.552 |
| p24 | 4 | 0.886 | 0.446 | 0.639 | 879 | 11.5 | 0.496 |

**Supplementary Table S5. The tree species abundance and diversity of secondary forest in the southern Taihang Mountains.**
